# Mitochondria-derived reactive oxygen species drive GANT61-induced mesothelioma cell apoptosis

**DOI:** 10.18632/oncotarget.2729

**Published:** 2015-01-16

**Authors:** Chuan Bian Lim, Cecilia M. Prêle, Svetlana Baltic, Peter G. Arthur, Jenette Creaney, D. Neil Watkins, Philip J. Thompson, Steven E. Mutsaers

**Affiliations:** ^1^ Lung Institute of Western Australia and Centre for Asthma, Allergy and Respiratory Research, School of Medicine and Pharmacology, University of Western Australia, Harry Perkins Institute of Medical Research, Nedlands, WA, Australia; ^2^ Centre for Cell Therapy and Regenerative Medicine, School of Medicine and Pharmacology, University of Western Australia and Harry Perkins Institute of Medical Research, Nedlands, WA, Australia; ^3^ School of Chemistry and Biochemistry, University of Western Australia, Crawley, WA, Australia; ^4^ National Centre for Asbestos Related Diseases, School of Medicine and Pharmacology, University of Western Australia, Harry Perkins Institute of Medical Research and Australian Mesothelioma Tissue Bank, Sir Charles Gairdner Hospital, Nedlands, WA, Australia; ^5^ Garvan Institute of Medical Research, Darlinghurst, NSW, Australia

**Keywords:** Apoptosis, mesothelioma, GANT61, Hedgehog, reactive oxygen species

## Abstract

Gli transcription factors of the Hedgehog (Hh) pathway have been reported to be drivers of malignant mesothelioma (MMe) cell survival. The Gli inhibitor GANT61 induces apoptosis in various cancer cell models, and has been associated directly with Gli inhibition. However various chemotherapeutics can induce cell death through generation of reactive oxygen species (ROS) but whether ROS mediates GANT61-induced apoptosis is unknown. In this study human MMe cells were treated with GANT61 and the mechanisms regulating cell death investigated. Exposure of MMe cells to GANT61 led to G1 phase arrest and apoptosis, which involved ROS but not its purported targets, GLI1 or GLI2. GANT61 triggered ROS generation and quenching of ROS protected MMe cells from GANT61-induced apoptosis. Furthermore, we demonstrated that mitochondria are important in mediating GANT61 effects: (1) ROS production and apoptosis were blocked by mitochondrial inhibitor rotenone; (2) GANT61 promoted superoxide formation in mitochondria; and (3) mitochondrial DNA-deficient LO68 cells failed to induce superoxide, and were more resistant to apoptosis induced by GANT61 than wild-type cells. Our data demonstrate for the first time that GANT61 induces apoptosis by promoting mitochondrial superoxide generation independent of Gli inhibition, and highlights the therapeutic potential of mitochondrial ROS-mediated anticancer drugs in MMe.

## INTRODUCTION

Malignant mesothelioma (MMe) is an aggressive fatal cancer, predominantly of the pleura and peritoneum, primarily caused by occupational asbestos exposure [1]. Current approaches involving surgery, radiotherapy or chemotherapy have failed to bring a clear survival benefit to patients, with a median survival of less than 12 months from diagnosis [2]. Clearly, new therapeutic strategies are urgently needed to improve the survival rate and quality of life of these patients.

The Hedgehog (Hh) pathway is a conserved signaling pathway responsible for the regulation of pattern specification during embryonic development [3]. It is also involved in the control of adult tissue homeostasis and stem cell maintenance [4]. For Hh signaling, the Hh protein acts as a ligand for the Patched1 (PTCH1) transmembrane receptor proteins [5]. PTCH1 interaction with transmembrane signal transducer Smoothened (SMO) under normal conditions inhibits SMO function [6]. Upon binding of Hh ligand to PTCH1, the inhibition of SMO by PTCH1 is alleviated, resulting in the activation of Gli transcription factors, capable of regulating expression of Hh target genes [7].

Several small molecule compounds with inhibitory effects on the Hh pathway have been reported. Notably, US FDA has approved GDC-0449, a SMO antagonist, for the treatment of patients with advanced stages of basal cell carcinoma [8]. Besides targeting SMO, the Hh ligand and Gli transcription factors appear highly amenable to inhibition by small molecule inhibitors. Of particular interest is GANT61, discovered in a cell-based screen for antagonists of Gli-mediated transcription [9]. GANT61 induces apoptosis in various human cancer cells including MMe [10–13] and suppresses the growth of prostate carcinoma [9] and neuroblastoma [14] xenografts in mice, suggesting that Gli proteins could be a therapeutic target in these cancers. The cytotoxic effect of GANT61 was previously attributed to the inhibition of Gli binding to DNA [9], thus preventing the transcription of Hh target genes such as Bcl-2 [10, 15], which are involved in cell proliferation and apoptosis. Recent studies showed that GANT61 induced autophagic death in hepatocellular carcinoma (HCC) cells through upregulation of Bnip3 [16]. However, it is unclear whether GANT61 have effects independent of Hh/Gli signaling.

Reactive oxygen species (ROS) are traditionally viewed as toxic molecules that damage cellular DNA, proteins and lipids [17]. However recently, ROS have been shown to have important physiological signaling functions [18]. Generation of ROS in response to chemo- and radio-therapy has been reported to induce cell cycle arrest and/or cell death in various cancer models [19]. However, whether GANT61 induces ROS generation has not been reported. Therefore, we examined the role of ROS in mediating GANT61-induced apoptosis and determined if this occurred via Hh/Gli signaling.

## RESULTS

### GANT61 induces G1 cell cycle arrest and apoptosis in human MMe cells

**Figure 1:**
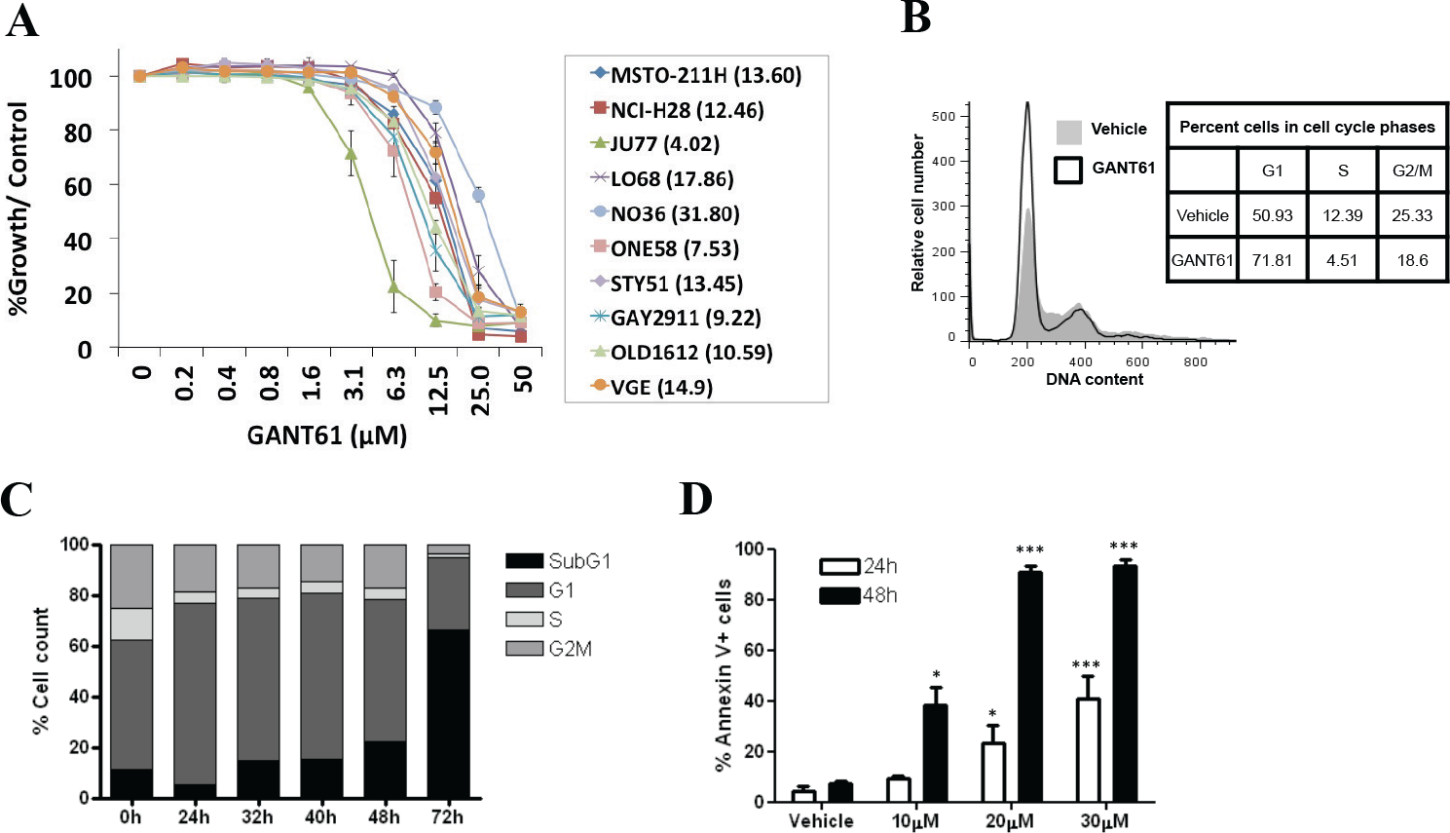
GANT61 induces G1 phase arrest and apoptosis in human MMe cells **(A)** Dose-response cytotoxicity curves for MMe cells treated with GANT61 for 72 h. The dose range tested was 0–50 μM. IC_50_ values are shown in brackets for each cell line. Values are the mean of independent experiments performed in 6 replicates (mean ± SEM; *n* = 3). **(B)** Cell cycle analysis of LO68 cells treated for 24 h with either 20 μM GANT61 (open graph) or vehicle (grey graph). The inset shows the percentage of cells at different phases of the cell cycle (G1, S and G2/M) of GANT61- and vehicle-treated cells. **(C)** Cell cycle analysis of LO68 cells treated for 0–72 h with 20 μM GANT61. **(D)** Apoptosis (as assessed by the annexin V/7AAD assay) was quantified in LO68 cells treated with 10, 20 or 30 μM GANT61 or vehicle for 24–48 h. Bar graphs show the quantification of results from independent experiments (mean ± SEM, *n* = 3). *, *p* < 0.05 or ***, *p* < 0.001, compared to vehicle-treated cells.

The antiproliferative activity of GANT61 was assessed in MMe cells. GANT61 inhibited cell proliferation in all MMe cell lines in a dose-dependent manner (Figure 1A). Among the cell lines tested, JU77 cells showed highest sensitivity to GANT61 treatment (IC_50_ = 4.02 μM) whereas NO36 cells appeared to be the most resistant (IC_50_ = 31.8 μM) (Figure 1A). As shown in Figure 1B, concomitant with growth inhibitory effect, GANT61 induced G1 cell cycle arrest as indicated by the increased percentage of LO68 cells in G1 cell cycle phase at 24 h compared to vehicle-treated cells. There was also an accumulation of cells in the sub-G1 fraction at 48–72 h compared to vehicle-treated cells, suggesting that apoptosis is involved (Figure 1C). To understand the cell death response to GANT61 in LO68 cells, cells were treated with GANT61 and phosphatidylserine externalization, a marker of early apoptosis, determined. GANT61 induced a parallel increase in apoptotic cell death in a dose- and time-dependent manner, as indicated by FACS analysis of annexin V-binding (Figure 1D).

### GANT61 targets Gli transcription factors in MMe cells

**Figure 2:**
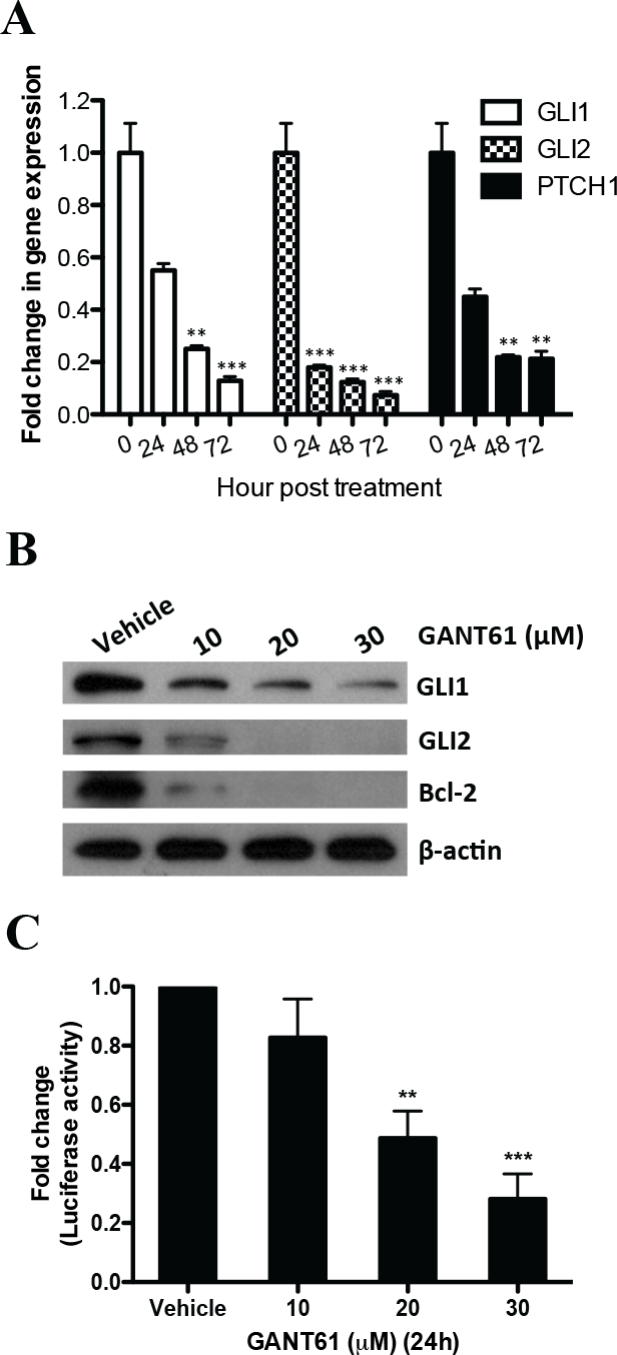
GANT61 targets Gli transcription factors in MMe cells **(A)** qRT-PCR analysis of the Hh pathway genes *GLI1*, *GLI2* and *PTCH1* was performed on LO68 cells treated with 20 μM GANT61 or vehicle for 24–72 h. The expression levels of each gene were normalized using *PGK1* mRNA as an endogenous control and are indicated as the fold change with respect to the vehicle-treated LO68 cells. Values represent the mean ± SEM of three independent experiments each performed in duplicate. **, *p* < 0.01 or ***, *p* < 0.001, compared to vehicle-treated cells. **(B)** Western blot analysis of GLI1, GLI2, Bcl-2 and β-actin on LO68 cells treated with 10, 20 or 30 μM GANT61 or vehicle for 24 h. **(C)** Gli transcriptional activity was determined by transfecting LO68 cells with a Gli-responsive luciferase reporter plasmid. Cells were treated with either 10, 20 or 30 μM GANT61 or vehicle for 24 h. Luciferase activity of cell lysates was measured and normalized to *Renilla* luciferase activity obtained by co-transfection with a constitutively expressed Renilla luciferase internal control plasmid. Results are expressed as the mean ± SEM from three independent experiments. **, *p* < 0.01 or ***, *p* < 0.001, compared to vehicle-treated cells.

GANT61 reduced mRNA expression of *GLI1* and *GLI2* following treatment with 20 μM GANT61 for up to 72 h (Figure 2A) as well as the Gli downstream target gene *PTCH1* (Figure 2A). A similar downregulation of GLI1 and GLI2 proteins was observed after 24 h exposure to different concentrations of GANT61 (10–30 μM) (Figure 2B). The protein level of Bcl-2, a GLI1 downstream target gene [20], was also reduced after GANT61 treatment (Figure 2B). To confirm the specificity of inhibition of GLI1 and GLI2 by GANT61, we tested its efficacy in a Gli luciferase reporter assay. Consistent with previous findings, GANT61 inhibited the Gli reporter activity in LO68 cells (Figure 2C). These findings point to GANT61 being an inhibitor of GLI1 and GLI2 [9].

### GANT61 induces oxidative stress

**Figure 3:**
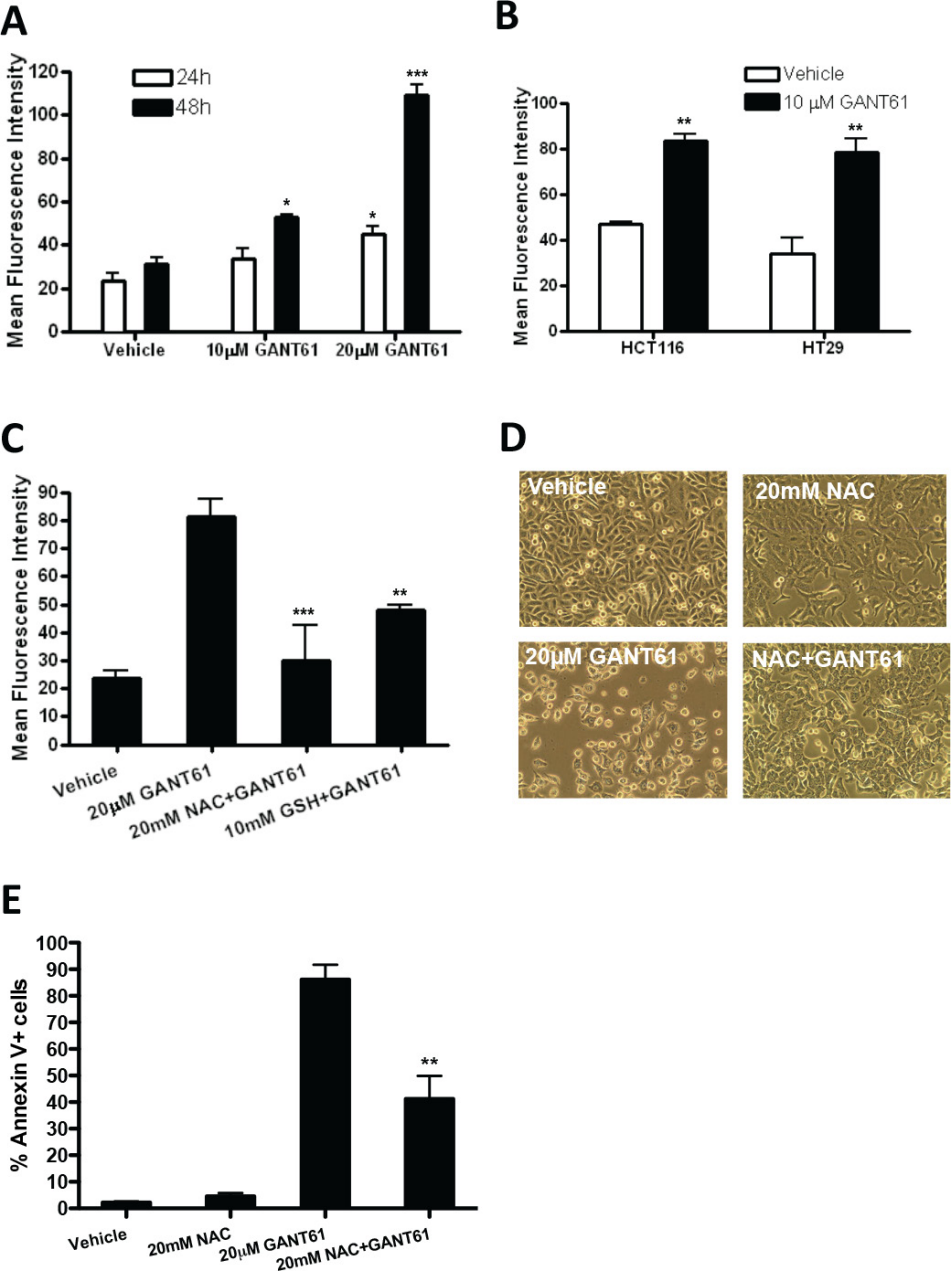
Oxidative stress is involved in GANT61-induced apoptosis **(A)** GANT61 treatment in LO68 cells is associated with an increase in ROS in a dose-and time-dependent manner. LO68 cells were treated with 10 and 20 μM GANT61 or vehicle for 24 or 48 h. The cells were stained with the fluorescent probe CH_2_DCFDA, and the fluorescence was measured by flow cytometry. Data are expressed as mean fluorescence intensity of positive cells measured in each experimental condition. Values are the average of independent measurements (mean ± SEM; *n* = 3). *, *p* < 0.05 or ***, *p* < 0.001, compared to vehicle-treated cells. **(B)** ROS is induced in GANT61-treated colon cancer cells. HCT116 and HT29 cells were treated with vehicle or GANT61 (10 μM) for 48 h before subjected to CH_2_DCFDA flow cytometric analysis. Values are the average of independent measurements (mean ± SEM; *n* = 3). **, *p* < 0.01, compared to vehicle-treated cells. **(C)** LO68 cells were treated with vehicle or 20 μM GANT61 for 48 h with or without the antioxidants NAC (20 mM) and GSH (10 mM). Bar graph shows the increase in the mean fluorescence intensity of CH_2_DCFDA-positive cells measured in each experimental condition. Values are the average of independent measurements (mean ± SEM; *n* = 3). **, *p* < 0.01 or ***, *p* < 0.001, compared to vehicle-treated cells. **(D)** Representative light micrographs showing the effects of 20 μM GANT61 or vehicle after 48 h with or without 20 mM antioxidant NAC on the morphology of LO68 cells. Note the reduced cell growth and altered cell morphology in GANT61-treated LO68 cells, which were not seen in vehicle-treated cells. Pretreatment with the antioxidant NAC restores cell growth and abrogates apoptosis in LO68 cells treated with 20 μM GANT61 for 24 h. **(E)**  Apoptosis (as assessed by the annexin V/7AAD assay) was quantified in LO68 cells treated with vehicle or GANT61 (20 μM) with or without 20 mM antioxidant NAC for 48 h. Bar graphs show results from independent experiments (mean ± SEM, *n* = 3). **, *p* < 0.01, compared to GANT61-treated cells.

Previous studies showed that GANT61 can induce DNA damage in colon cancer cells [10]. We hypothesize that GANT61 triggers the production of reactive oxygen species (ROS), which in turn damages DNA. To test this hypothesis, cells were treated with GANT61 (10–20 μM) for 24 to 48 h and intracellular ROS levels were measured using the carboxy derivative of fluorescein, CH_2_DCFDA. As shown in Figure 3A, ROS levels increased significantly in LO68 cells treated with GANT61 in a dose- and time-dependent manner. GANT61 also triggered ROS generation in HCT116 and HT29 colon cancer cells, suggesting that the production of ROS could be a general effect of GANT61 exposure (Figure 3B). Moreover, pretreatment of LO68 cells with N-acetylcysteine (NAC) and reduced L-glutathione (GSH), two potent ROS scavengers, attenuated this accumulation of ROS (Figure 3C). As shown in Figure 3D, neutralization of ROS by NAC in GANT61-treated cells restored cell viability, suggesting that ROS is responsible for GANT61 cytotoxicity. Consistent with this data, annexin V/7AAD assays showed that NAC pretreatment rescued LO68 cells from GANT61-induced apoptosis (Figure 3E).

### GANT61 downregulates GLI1, GLI2 and PTCH1 through ROS

**Figure 4:**
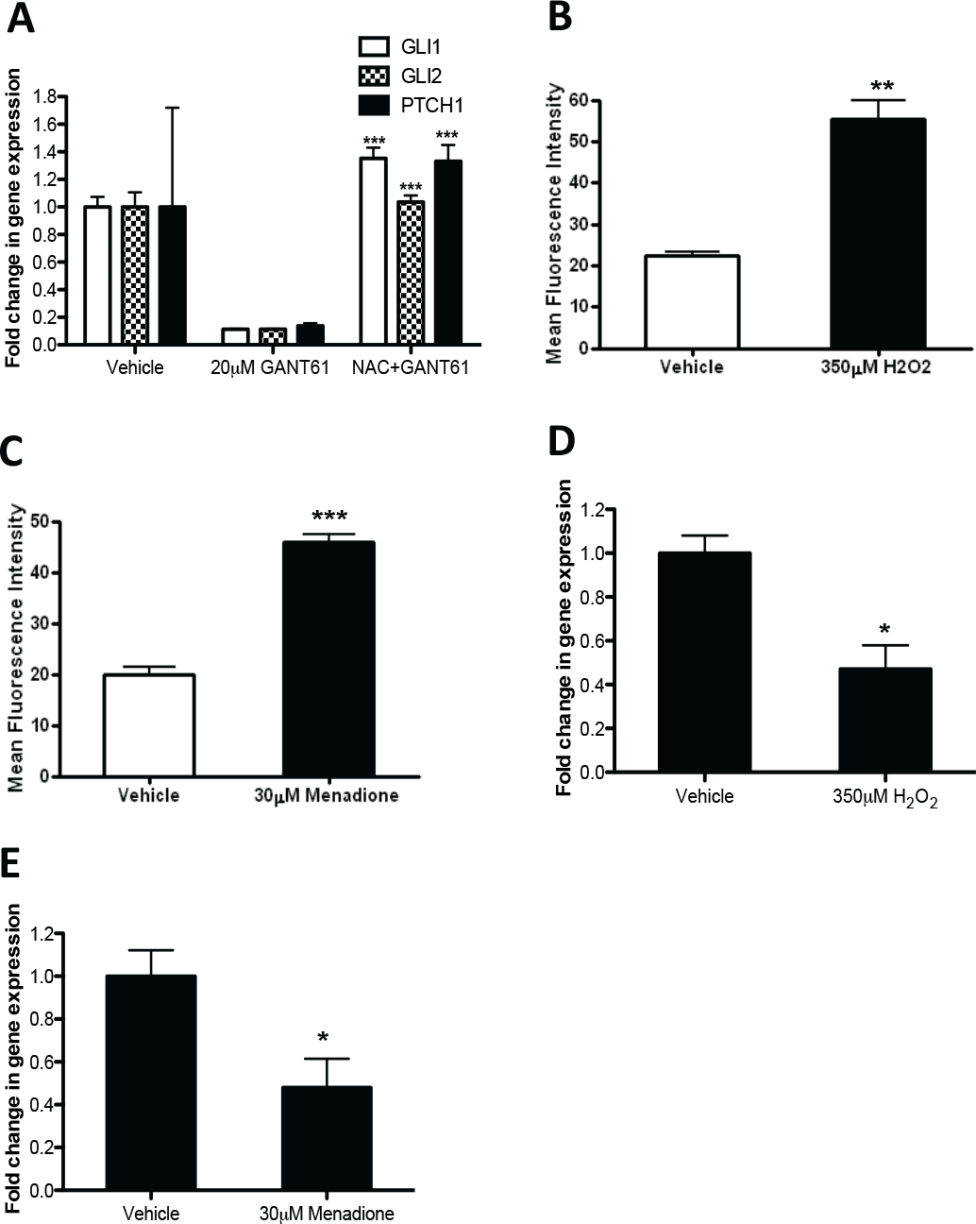
GANT61 downregulates GLI1, GLI2 and PTCH1 through ROS **(A)** Analyses of Hh pathway genes (*GLI1*, *GLI2* and *PTCH1*) by qRT-PCR. LO68 cells were pretreated with NAC for 1 h and then with 20 μM GANT61 or vehicle for 48 h. Values represent the mean ± SEM of three independent experiments each performed in duplicate. ***, *p* < 0.001, compared to GANT61-treated cells. **(B)** LO68 cells were treated with vehicle or 350 μM H_2_O_2_ for 24 h. The cells were stained with CH_2_DCFDA and the fluorescence measured by flow cytometry. Bar graph represents the increase in mean fluorescence intensity of positive cells measured in each experimental condition. Values are the average of independent measurements (mean ± SEM; *n* = 3). **, *p* < 0.01, compared to vehicle-treated cells. **(C)** LO68 cells were treated with vehicle or 30 μM menadione for 24 h. The cells were stained with CH_2_DCFDA and fluorescence measured by flow cytometry. Bar graph shows the increase in the mean fluorescence intensity of positive cells measured in each experimental condition. Values are the average of independent measurements (mean ± SEM; *n* = 3). ***, *p* < 0.001, compared to vehicle-treated cells. **(D)** Analysis of *GLI1* mRNA expression by qRT-PCR. LO68 cells were treated with vehicle or 350 μM H_2_O_2_ for 24 h. Values represent the mean ± SEM of three independent experiments each performed in duplicates. *, *p* < 0.05, compared to vehicle-treated cells. **(E)** Analysis of *GLI1* mRNA expression by qRT-PCR. LO68 cells were treated with vehicle or 30 μM menadione for 24 h. Values represent the mean ± SEM of three independent experiments each performed in duplicates. *, *p* < 0.05, compared to vehicle-treated cells.

We next examined the effect of NAC on GANT61-mediated *GLI1*, *GLI2* and *PTCH1* expression. As shown in Figure 4A, the downregulation of *GLI1*, *GLI2* and *PTCH1* expression by GANT61, as determined by qRT-PCR, was abolished by pretreating cells with NAC. The blockade of ROS accumulation by NAC prevents reduction of *GLI1*, *GLI2* and *PTCH1* expression indicating the involvement of ROS in modulation of the Hh pathway. Stimulated by our novel finding that ROS could potentially impact on the Hh pathway, we next assessed the effect of exposing LO68 cells to menadione, a ROS generator, and hydrogen peroxide (H_2_O_2_), a mimic of oxidative stress, on the Hh pathway. FACS analysis of intracellular ROS production indicated that exposure of LO68 cells to menadione and H_2_O_2_ resulted in a significant increase in ROS production as measured by the fluorescent CH_2_DCFDA probe (Figure 4B and 4C). Furthermore, qRT-PCR analysis of gene expression in LO68 cells following treatment clearly indicated the ability of menadione and H_2_O_2_ to downregulate the expression of *GLI1*, a marker of Hh pathway activity (Figure 4D and 4E). Together, these findings suggest that ROS plays a critical role in the suppression of *GLI1*, *GLI2* and *PTCH1* expression by GANT61.

### GANT61-induced ROS, apoptosis and cell cycle arrest are independent of Gli inhibition

**Figure 5:**
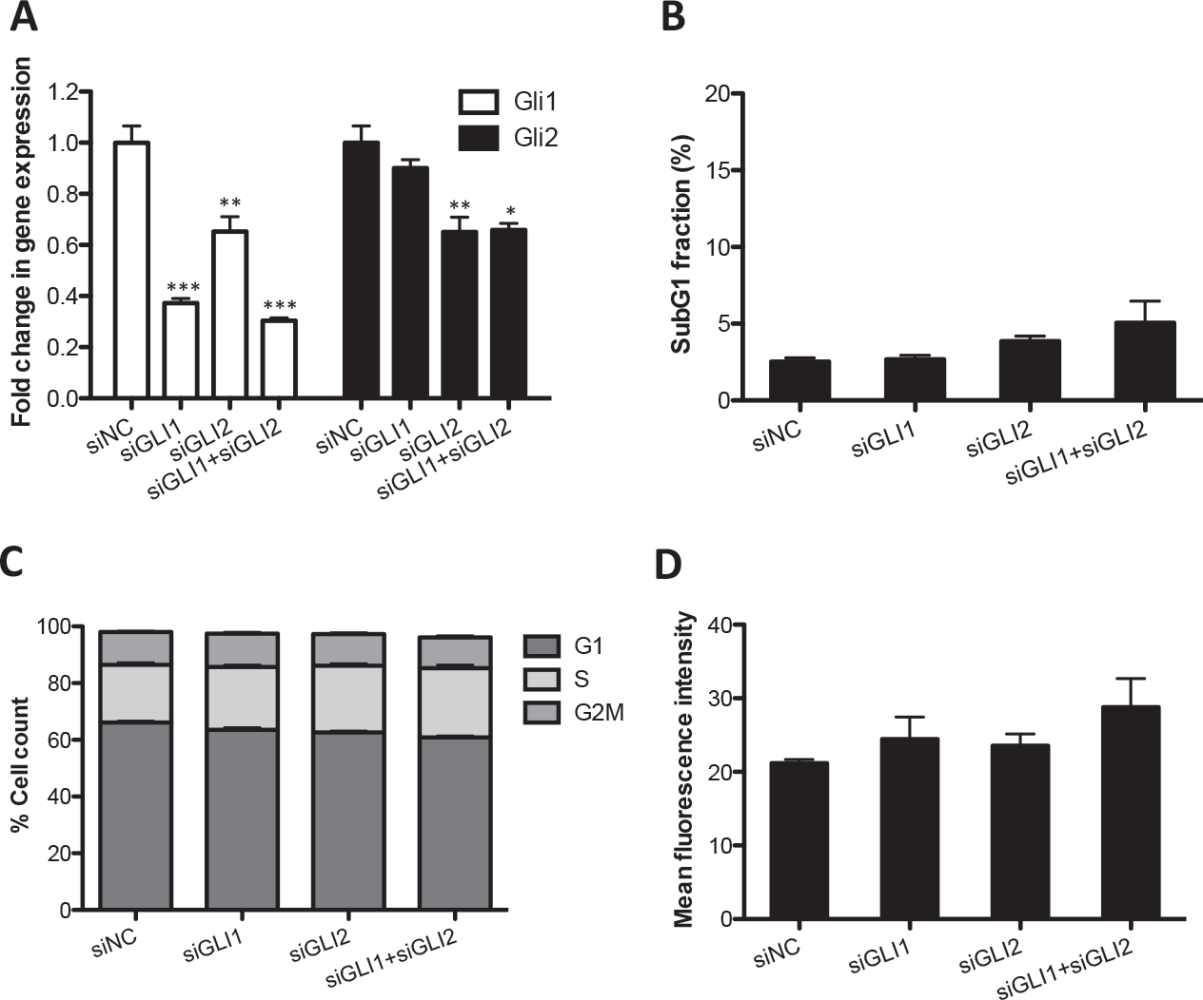
Knockdown of GLI1 and GLI2 by siRNA does not increase ROS production in MMe cells **(A)** LO68 cells were transfected with negative control (siNC), GLI1 (siGLI1) or GLI2 (siGLI2) siRNA for 96 h, then *GLI1* mRNA expression analyzed by qRT-PCR. Values represent the mean ± SEM of three independent experiments each performed in duplicates. *, *p* < 0.05, **, *p* < 0.01 or ***, *p* < 0.001, compared to NC siRNA-transfected cells. **(B)** Cell death (subG1 fraction) was measured by flow cytometry 96 h after transfection. Data represent the mean ± SEM of three independent experiments. **(C)** The cell cycle was analyzed by flow cytometry 96 h following transfection. Histogram profiles of flow-cytometric analysis show the cell cycle distribution of the cell population. Data represent the mean ± SEM of three independent experiments. **(D)** The level of intracellular ROS was monitored using CH_2_DCFDA and the fluorescence measured by flow cytometry 96 h following transfection. Data represent the mean ± SEM of three independent experiments.

Because GANT61 is an inhibitor of Gli transcription factors, we set out to determine whether GANT61-induced G1 cell cycle arrest, apoptosis and ROS production are dependent on Gli inhibition. First, we examined apoptosis and ROS production in LO68 cells following siRNA-mediated depletion of GLI1 and GLI2. qRT-PCR analyses demonstrated knockdowns of GLI1 and GLI2 mRNA in LO68 cells (Figure 5A). Surprisingly however, neither individual GLI1 or GLI2 knockdowns nor their combined depletion induced cell death as indicated by the small sub-G1 apoptotic population (< 5% in sub-G1 phase in GLI1- and/or GLI2-depleted cells compared to ~5% cells in sub-G1 phase in LO68 cells transiently transfected with negative control (NC) siRNA), indicating that the pro-apoptotic activity of the drug is likely to be independent of Gli inhibition (Figure 5B). Similarly, the results from FACS analysis showed that the knockdowns of GLI1 and GLI2 by siRNA did not result in G1 cell cycle arrest (~60% in G1 phase in GLI1- and/or GLI2-depleted cells compared to ~60% cells in G1 phase in LO68 cells transiently transfected with NC siRNA) (Figure 5C). Moreover, depletion of GLI1 and GLI2, individually or together using siRNAs did not result in ROS generation in LO68 cells (Figure 5D). Taken together, our data suggest that generation of ROS and induction of apoptosis and cell cycle arrest in response to GANT61 is independent of Hh/Gli signaling in LO68 cells.

### GANT61-induced apoptosis and ROS production is dependent on mitochondria

**Figure 6:**
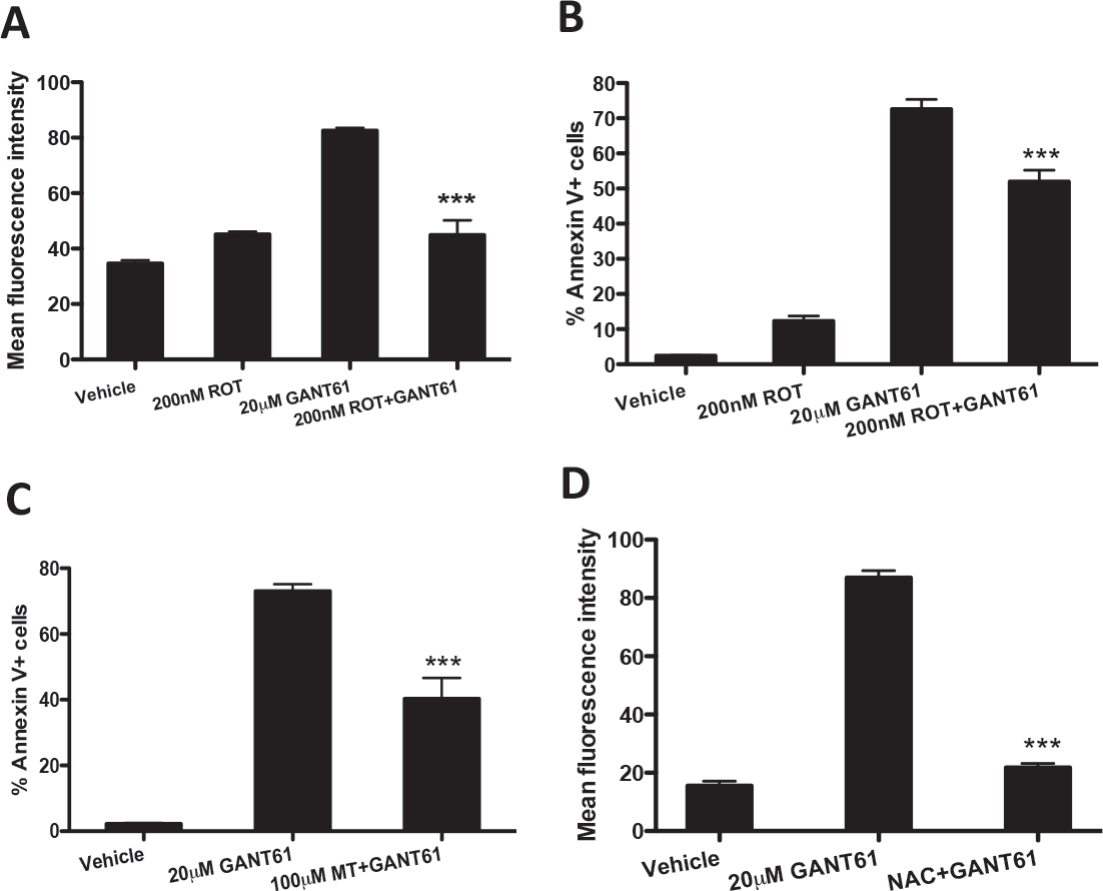
GANT61-induced apoptosis and ROS production is dependent on mitochondria **(A)** LO68 cells were pretreated with 200 nM rotenone (ROT), an inhibitor of mitochondrial complex 1, for 1 h, and further incubated with 20 μM GANT61 for 48 h. The level of intracellular ROS was monitored using CH_2_DCFDA and the fluorescence measured by flow cytometry. Data represent the mean ± SEM of three independent experiments. ***, *p* < 0.001, compared to GANT61-only treated cells. **(B)** Apoptosis was measured by annexin V/7AAD assay. Data represent the mean ± SEM of three independent experiments. ***, *p* < 0.001, compared to GANT61-only treated cells. **(C)** Mitochondria-targeted superoxide dismutase mimetic mitoTEMPO (MT) attenuates GANT61-induced apoptosis. LO68 cells were pretreated with MT (100 μM) for 1 h and further incubated with 20 μM GANT61 for 48 h. Apoptosis was measured by annexin-V/7AAD assay. Data represent the mean ± SEM of three independent experiments. ***, *p* < 0.001, compared to GANT61-only treated cells. **(D)** GANT61 induces mitochondrial superoxide production in LO68 cells. Cells were pretreated with NAC (20 mM) for 1 h and further treated with 20 μM GANT61 or vehicle for 48 h before subjected to mitoSOX red flow cytometric analysis. Bar graph represents the increase in the mean fluorescence intensity of mitoSOX red-positive cells measured in each experimental condition. Values are the average of independent measurements (mean ± SEM; *n* = 3). ***, *p* < 0.001, compared to GANT61-treated cells.

The mitochondrion is a major site of ROS generation in mammalian cells [18]. To determine the site of ROS production in response to GANT61, LO68 cells were treated with GANT61 in the absence or presence of rotenone, a mitochondrial complex I inhibitor, and the effects on GANT61-induced ROS generation and apoptosis were assessed. GANT61-induced ROS production (Figure 6A) and apoptosis (Figure 6B) were significantly blocked by the addition of rotenone, indicating that the ROS produced in response to GANT61 was of mitochondrial origin. The addition of a mitochondria-targeted antioxidant, MitoTEMPO, also blocked the increase in apoptosis induced by GANT61 (Figure 6C). We further confirm the mitochondrial origin of ROS by measuring the levels of superoxide within mitochondria after exposure to GANT61 using the fluorescent probe mitoSOX red. As shown in Figure 6D, exposure to 20 μM GANT61 for 48 h induced an increase in intramitochondrial superoxide levels. In addition, this increase in intramitochondrial superoxide was attenuated by pretreating cells for 1 h with 20 mM NAC before co-treating with 20 μM GANT61 (Figure 6D).

### Mitochondrial superoxide is essential for GANT61-induced apoptosis

**Figure 7:**
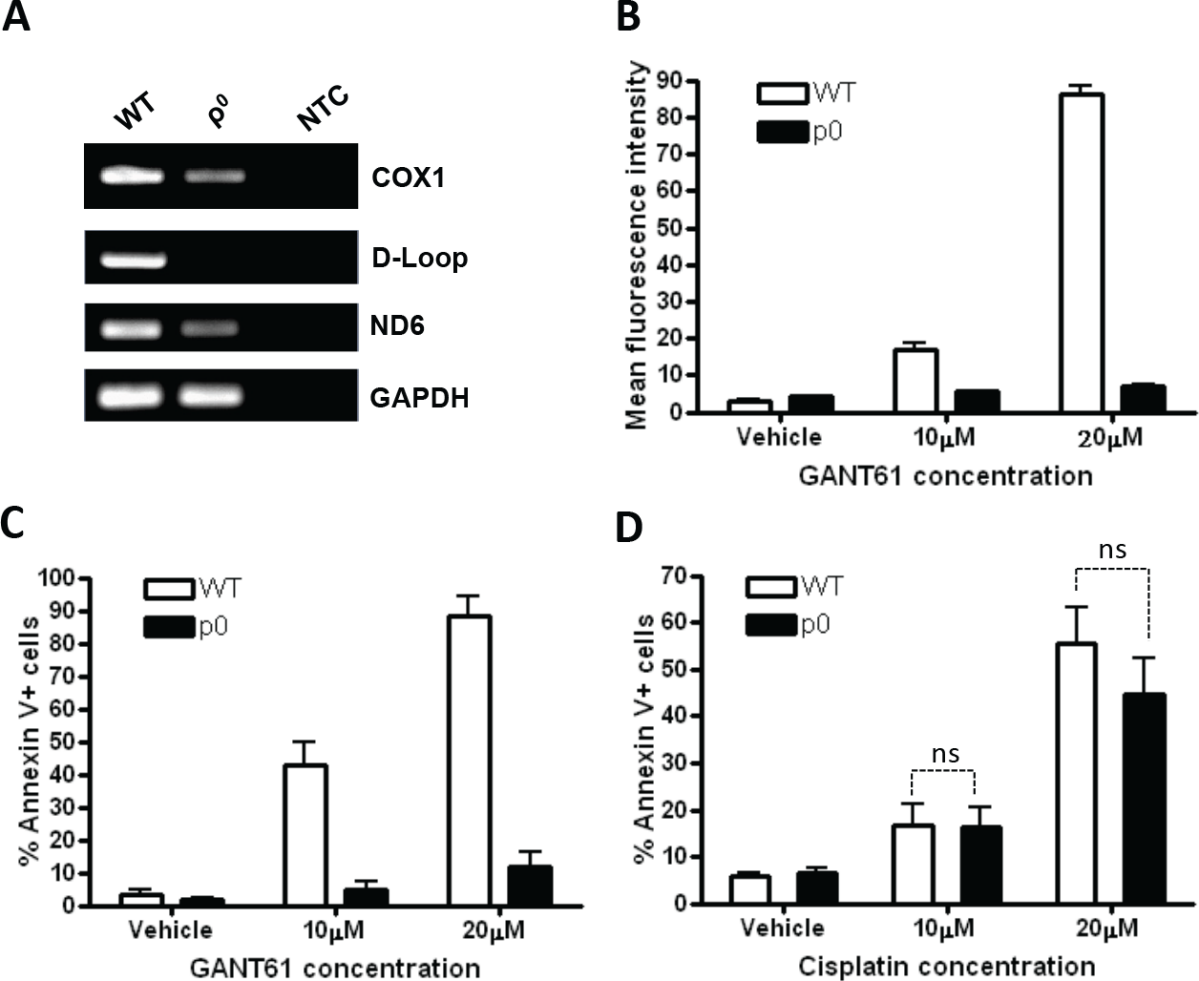
Mitochondrial ROS is required for GANT61-mediated apoptosis **(A)** Depletion of mitochondrial DNA in LO68 cells. Total DNA (20 ng) from wild-type (WT) and mitochondrial DNA-depleted ρ^0^ LO68 cells were subjected to PCR amplification using primers that were designed from specific regions of the mitochondrial DNA coding for *COX1*, *D-loop* and *ND6*. *GAPDH* was included as a control for nuclear DNA-encoded gene. NTC, no template control. **(B)** Detection of mitochondrial superoxide on GANT61 treatment. WT or ρ^0^ LO68 cells were treated with 10–20 μM GANT61 or vehicle for 48 h. The cells were then stained with mitoSOX red, and the fluorescence was measured by flow cytometry. Bar graph represents the increase in the mean fluorescence intensity of mitoSOX red-positive cells measured in each experimental condition. Values are the average of independent measurements (mean ± SEM; *n* = 3). **(C)** ρ^0^ LO68 cells are resistant to GANT61-induced apoptosis. WT or ρ^0^ LO68 cells were treated with 10–20 μM GANT61 for 48 h. Apoptosis was then measured by annexin-V/7AAD assay. Data represent the mean ± SEM of three independent experiments. **(D)** No apparent difference was observed in the sensitivity to apoptosis by cisplatin in WT or ρ^0^ LO68 cells. Cells were treated with 10–20 μM Cisplatin for 48 h. Apoptosis was then measured by annexin-V/7AAD assay. Data represent the mean ± SEM of three independent experiments. ns, not significantly different from untreated control cells.

To genetically confirm involvement of mitochondrial superoxide in GANT61-induced apoptosis, we generated LO68 ρ^0^ cells, which lack mitochondrial DNA, by exposing cells to low concentration of ethidium bromide. Mitochondrial DNA depletion was verified in LO68 ρ^0^ cells by amplifying three mitochondria-encoded genes, *COX1*, *D-loop* and *ND6*, by PCR. In addition, *GADPH* was amplified to serve as a control for nuclear-encoded genes. As shown in Figure 7A, ethidium bromide treatment resulted in a marked reduction of PCR products for *COX1*, *D-loop* and *ND6*. There was no difference in the levels of *GAPDH* PCR product between LO68 and LO68 ρ^0^ cells, indicating that nuclear DNA was not depleted in the process of establishing LO68 ρ^0^ cells (Figure 7A). Next, LO68 and LO68 ρ^0^ cells were treated with GANT61 (10–20 μM) for 48 h before staining with mitoSOX red. In LO68 cells, exposure to GANT61 for 48 h resulted in an induction of mitochondrial superoxide formation. MitoSOX red oxidation was significantly reduced in LO68 ρ^0^ cells, indicating that a functional respiratory chain is required for the GANT61 induction of superoxide formation (Figure 7B). To determine whether functional mitochondrial respiratory chain is important for mediating GANT61-induced apoptosis, we compared apoptosis in LO68 and LO68 ρ^0^ cells exposed to GANT61 (10 and 20 μM) for 48 h. As shown in Figure 7C, GANT61 increased apoptosis in a dose-dependent manner in LO68 cells as assessed by annexin V staining. In contrast, GANT61 induced negligible apoptosis in LO68 ρ^0^ cells after exposure to GANT61. Furthermore, to exclude the possibility that LO68 ρ^0^ cells were resistant to apoptosis because of their loss of a functional mitochondrial respiratory chain, LO68 and LO68 ρ^0^ cells were treated with cisplatin (10 and 20 μM), a standard chemotherapeutic drug known to induce apoptosis in LO68 cells, for 48 h, and apoptosis was assessed by annexin V staining. As shown in Figure 7D, LO68 and LO68 ρ^0^ cells undergo apoptosis when treated with cisplatin. Also there was no statistical difference in the level of apoptosis between LO68 and LO68 ρ^0^ cells as demonstrated by annexin V assay (*p* > 0.05). Taken together, these results suggest that GANT61-induced apoptosis is mediated by mitochondrial superoxide.

## DISCUSSION

Targeting the Hh pathway either through RNA-interference knockdown of GLI1 and GLI2 or using Gli inhibitors, has been shown to induce growth inhibition and cell death in mesothelioma cells and xenograft tumors *in vivo* [13, 21, 22]. A promising anticancer agent GANT61, with Gli inhibitory activity, displayed potent cytotoxic activity against diverse human cancer types including MMe [13–15, 23–25]. On the basis of computational docking and surface plasmon resonance data, GANT61 has been proposed to mediate its pro-apoptotic effect by binding directly to GLI1, which in turn inhibits GLI1 from binding to DNA [9, 26]. There is evidence that GANT61 appears to have a novel anticancer mechanism that differs from other Hh antagonists. Recent studies have reported that GANT61 triggers apoptosis via induction of DNA double strand breaks and activation of ATM-Chk2 DNA damage response in colon cancer cells [10, 27].

Using a panel of MMe cell lines, we have demonstrated that GANT61-induced anti-proliferative effects were related to the inhibition of cell growth, as confirmed by reduction of cell growth and induction of G1 cell cycle arrest. Our data also show that GANT61 induced apoptotic cell death in LO68 cells in a concentration- and time-dependent manner. We present data indicating that GANT61 specifically acts on the Hh-Gli pathway, as demonstrated by a reduction in the expression levels of downstream pathway effectors GLI1 and GLI2 and Gli target genes PTCH1 and Bcl-2. GANT61 also significantly decreased the Gli-luciferase reporter activity in a dose-dependent manner. These results are consistent with previous reports in HEK294 cells transiently overexpressing GLI1 and colon cancer cells with constitutively active Hh signaling [9, 15]. At this juncture, our data corroborate previous findings that GANT61 inhibits Hh signaling at the level of Gli transcription factors [9, 14, 15]. However, silencing GLI1 and GLI2, individually or together, were not sufficient to induce cell death in LO68 cells, strongly suggesting that GANT61-induced apoptosis was not associated with Gli inhibition. Our data is in contrast to previous siRNA experiments, where silencing of GLI1 resulted in increased apoptosis and reduced level of anti-apoptotic Bcl-2 in HCC, glioma and breast cancer cells [28–30]. Thus, it is possible that GANT61-induced apoptosis may be initiated by another factor other than Gli inhibition.

An alternative explanation for Gli-independent induction of apoptosis by GANT61 is production of ROS. Previous reports have shown that certain chemotherapeutic drugs can induce caspase-independent apoptosis that is brought about by the production of ROS [31, 32]. The present studies showed that ROS were generated concomitantly with apoptosis in a dose- and time-dependent manner in LO68 cells upon treatment of cells with GANT61. Interestingly, similar to GANT61-induced apoptosis, Gli silencing by siRNA showed that although GLI1 and GLI2 were downregulated in LO68 cells, they did not appear to be involved in induction of ROS generation. Furthermore, the apoptogenic role of ROS production was supported by the ability of two general antioxidants, NAC and GSH, to rescue cells from GANT61-induced apoptosis. However, it is hard to determine the specific ROS species that are generated from GANT61 exposure in LO68 cells. It seems likely that the superoxide produced in the mitochondria might play an important role in the induction of apoptosis. Approximately 1–2% of electrons can “leak” to oxygen to form superoxide in a reaction mediated mainly by complex I and III of the mitochondrial respiratory chain [33]. To identify the source of ROS, rotenone, a complex I inhibitor, was able to reduce GANT61-induced ROS production and rescue LO68 cells from GANT61-induced apoptosis when it was added to cells prior to GANT61 exposure. This result implied that GANT61-induced ROS might come from mitochondria. Because the blockade of GANT61-induced apoptosis by rotenone was partial, there could also be other sites of ROS production. Another major site of ROS generation is plasma membrane NADPH oxidase [34].

Mitochondrial DNA-depleted ρ° cells lack a working respiratory chain and are not able to generate ATP and ROS within mitochondria [35, 36]. We thus hypothesized that the depletion of mitochondrial DNA in LO68 ρ° cells may interfere with ROS production and in turn apoptosis, after treatment with GANT61. Consistent with our hypothesis, LO68 ρ° cells showed resistance to GANT61, and lower mitochondrial superoxide levels were also observed after GANT61 treatment. The resistance of LO68 ρ° cells to GANT61 was not associated with a decreased susceptibility to apoptosis, as indicated by the equal sensitivity to cisplatin of wild-type and ρ° cells. Overall, our results are consistent with the hypothesis that susceptibility to GANT61 stems from exaggerated production of ROS from the mitochondrial respiratory chain.

**Figure 8:**
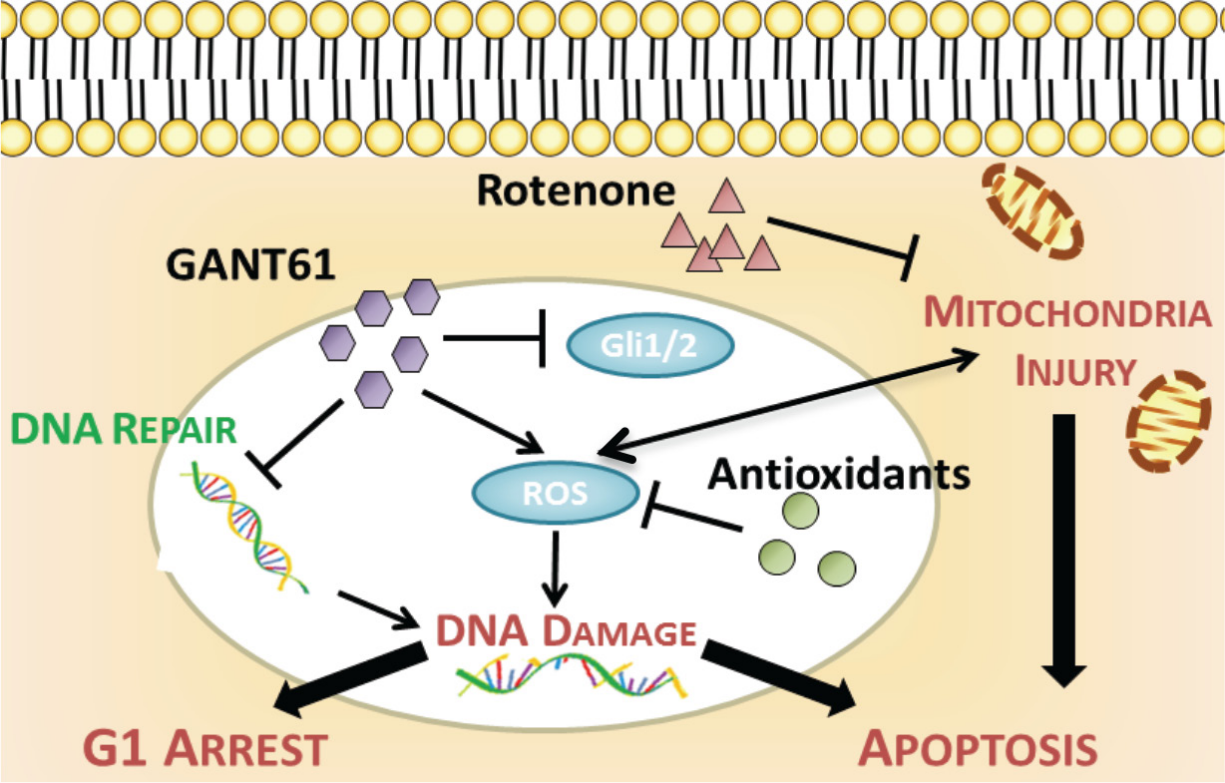
Schematic representation of the proposed mechanism of GANT61-induced apoptosis in MMe cells GANT61 triggers the production of mitochondrial ROS independent of Hh/Gli signaling. GANT61-induced ROS causes DNA damage and reduced DNA repair, which leads to G1 cell cycle arrest and apoptosis. Pretreatment of cells with either antioxidants or rotenone can reverse this ROS-induced apoptosis.

In conclusion, the present study not only demonstrates the therapeutic potential of GANT61 in MMe, but also demonstrates a novel mechanism for GANT61-induced G1 phase arrest and apoptosis in MMe cells. This is the first report of apoptosis induced by GANT61 via generation of mitochondrial ROS. Based on the findings in this paper, we propose a model showing the relationship of GANT61, ROS and mitochondria in the induction of growth suppression and apoptosis of MMe cells (Figure 8). Our results offer an initial proof-of-concept that mitochondrial ROS-mediated anticancer mechanisms may be exploited for therapeutic benefits in MMe.

## MATERIAL AND METHODS

### Cell lines and culture conditions

The cell lines MSTO-211H, NCI-H28, HCT116 and HT29 were obtained from the American Type Culture Collection; JU77, LO68, NO36, ONE58 and STY51 were gifts from Professor Bruce W. Robinson [37]; GAY2911, OLD1612 and VGE were derived from pleural fluids or tumors of MMe patients. Cells were cultured in Dulbecco's modified Eagle's medium supplemented with 10% fetal bovine serum (Serana), 4 mM L-glutamine (Life Technologies), 100 units/ml penicillin (Life Technologies) and 100 μg/ml streptomycin (Life Technologies) in a humidified 37°C incubator with 5% CO_2_.

### TaqMan quantitative real-time PCR analysis (qRT-PCR)

qRT-PCR was performed as described previously [38]. mRNA expression of genes was quantified using the following TaqMan gene expression arrays (Applied Biosystems): *GLI1* (hs01110766_m1), *GLI2* (hs01119974_m1), *PTCH1* (hs00181117_m1) and *PGK1* (4326318E-1006008). The levels of each gene were normalized to *PGK1* mRNA and presented as fold change with respect to untreated cells for each gene.

### Cell proliferation assay

Cell proliferation was determined using the methylene blue assay as previously described [39]. Briefly, cells were seeded in 96-well plates and treated the following day with GANT61 (0.2 – 50 μM) (Tocris Bioscience) or dimethyl sulfoxide (DMSO) (Sigma Aldrich) as vehicle control. Following treatment, cells were fixed with 4% paraformaldehyde (Sigma Aldrich) for 10 min at 4°C followed by staining with 2% methylene blue/0.01M borate (pH 8.5) solution (Sigma Aldrich). Excess dye was then washed off using 0.01M borate buffer (pH 8.5) and the methylene blue dye from cells were extracted with 1:1 (v/v) ethanol and 0.I M hydrochloric acid and quantitated at 650 nm on a Wallac 1420 VICTOR2 multilabel plate reader (Perkin Elmer). Half maximal inhibitory concentrations (IC_50_) were determined using Graphpad Prism 4.03 software (Graphpad Software, Inc.).

### Cell cycle analysis by DNA content

Cells were treated with 20 μM GANT61 for 24–72 h then fixed with 70% ethanol at 4°C. Cells were stained with 50 μg/ml propidium iodide (Sigma Aldrich) in the presence of 100 μg/ml DNase-free RNase A (Life Technologies) for 30 min at room temperature. Stained cells were analyzed for DNA content using a FACSCalibur flow cytometer (BD Biosciences) and quantified using the FlowJo software (Tree Star, Inc.).

### Detection of apoptosis

Detection of apoptotic cells was performed with the PE Annexin V Apoptosis Detection kit (BD Biosciences) according to the manufacturer's protocol. Briefly, cells were harvested after drug treatment, washed twice with ice-cold phosphate buffered saline (PBS) and incubated with Annexin V PE conjugate and 7-aminoactinomycin D (7AAD) for 15 min in the dark. Stained cells were analyzed by flow cytometry and quantified using the FlowJo software.

### ROS detection

Intracellular ROS production was determined by loading cells with 20 μM 6-carboxy-2′,7′-dichlorodihydrofluorescein diacetate (CH_2_DCFDA) (Life Technologies) at 37°C for 45 min. Intramitochondrial superoxide production was determined by loading cells with 5 μM mitoSOX red mitochondrial superoxide indicator (Life Technologies) at 37°C for 45 min. Red and green fluorescence emissions were analyzed by flow cytometry using excitation/emission wavelengths of 488/530 nm and 488/585 nm for CH_2_DCFDA and mitoSOX red, respectively.

### RNA interference

Cells were grown to 80% confluence and transfected with 100 nM GLI1 siRNA (Santa Cruz Biotechnology), GLI2 siRNA (Sigma Aldrich) or negative control siRNA (Santa Cruz Biotechnology) using Lipofectamine 2000 transfection reagent (Life Technologies) according to the manufacturer's instructions.

### Gli luciferase reporter assay

Gli transcriptional activity was measured using a Cignal Gli Reporter (luc) kit (SABiosciences) according to manufacturer's instructions. Briefly, cells were seeded in 12-well plates 24 h before transfection. Cells were cotransfected with 500 ng of Gli luciferase reporter construct and a Renilla luciferase construct (40:1 ratio) using Lipofectamine 2000 transfection reagent, with a 9:1 ratio (v/w) of Lipofectamine 2000 to DNA. Cells were harvested using the Dual-Glo Luciferase assay system (Promega) 48 h after transfection according to the manufacturer's instruction. Luciferase activity was measured using a microplate reader. All reporter assays were normalized to Renilla luciferase activity.

### Western blot analysis

Cells were harvested, lysed using CelLytic M mammalian cell lysis/extraction reagent (Sigma Aldrich), and the protein concentrations determined by NanoDrop 2000c Spectrophotometer (Thermo Scientific). Proteins (30 μg) were separated by sodium dodecyl sulfate-polyacrylamide gel electrophoresis and electrotransferred onto nitrocellulose membrane (Millipore), which was then blocked with 5% nonfat milk or bovine serum albumin (Sigma Aldrich) in 0.1% Tris-buffered saline-Tween 20 for 1 h at room temperature. The membranes were incubated at 4°C overnight with the indicated primary antibodies. Antibodies recognizing GLI1, GLI2 and Bcl-2 were purchased from Cell Signaling Technology. As a protein loading control, membranes were probed with a mouse anti-β-actin monoclonal antibody (Santa Cruz Biotechnology). After repeated washing to remove unbound antibodies, the membranes were further incubated with horseradish peroxide-conjugated anti-rabbit or anti-mouse secondary antibodies (Cell Signaling Technology) for 1 h at room temperature. Chemiluminescent detection of antibody binding was performed using the Immobilon Western HRP Substrate (Millipore).

### Generation of LO68 θ^0^ cells

LO68 ρ^0^ cells were generated by treating LO68 cells with 100 ng/ml ethidium bromide (Sigma Aldrich), 50 μg/ml uridine (Sigma Aldrich) and 1 mM sodium pyruvate (Life Technologies).

### PCR amplification

Total DNA was isolated from cells using the PureLink Genomic DNA kit (Life Technologies), according to the manufacturer's instructions. PCR primers that amplify genes coding for cytochrome c oxidase subunit 1 (*COX1*), *D-loop*, NADH dehydrogenase 6 (*ND6*) and glyceraldehyde-3-phosphate dehydrogenase (*GADPH*) were obtained from published literature [40, 41]. PCR amplification was performed as described [41] and the PCR products were visualized on 1.5% agarose gels.

### Statistical analysis

Statistical calculations were performed using Graphpad Prism 5 software (Graphpad Software, Inc.). Student's *t*-test was used to determine statistical differences between two groups. Statistical differences between multiple groups were calculated using one-way ANOVA analysis and Tukey's multiple comparison post-hoc test. *P* < 0.05 was considered statistically significant.
